# Cancer-associated fibroblasts in cholangiocarcinoma: the central nexus of tumor-stroma crosstalk and therapeutic translation

**DOI:** 10.3389/fimmu.2025.1725948

**Published:** 2025-12-09

**Authors:** Shaozhen Rui, Yuqi Liu, Yongqing Zhao, Xiaoliang Zhu, Shanhui Liao, Wence Zhou

**Affiliations:** 1 The Second Clinical Medical College, Lanzhou University, Gansu, Lanzhou, China; 2 Department of General Surgery, the First Hospital of Lanzhou University, Gansu, Lanzhou, China; 3 The Clinical Research Center for General Surgery of Gansu Province, Lanzhou, China; 4 The Cuiying College, Lanzhou University, Gansu, Lanzhou, China; 5 Department of General Surgery, the Second Hospital of Lanzhou University, Gansu, Lanzhou, China; 6 Key Laboratory of Environmental Oncology of Gansu Province, Lanzhou, China

**Keywords:** cholangiocarcinoma, cancer-associated fibroblasts, heterogeneity, interactions, targeted therapy

## Abstract

Cholangiocarcinoma (CCA) is a highly invasive malignant tumor of the biliary tract, and its detection is commonly delayed until advanced stages owing to a lack of early symptoms, with dismal overall survival and a high propensity for chemoresistance. CCA is primarily classified based on its anatomical location, encompassing distinct molecular subtypes with both intertumoral and intratumoral heterogeneity. Beyond malignant epithelial cells, CCA harbors a complicated and dynamically evolving tumor microenvironment (TME), in which multiple stromal cell types orchestrate disease progression through intricate crosstalk networks. Among them, cancer-associated fibroblasts(CAFs) constitute the numerically predominant cellular component in the matrix of CCA, playing pivotal roles in extracellular matrix remodeling, immune regulation, angiogenesis, and metastasis. Traditionally regarded as predominantly tumor-promoting, CAFs have recently been recognized as a heterogeneous population with transcriptionally and functionally distinct subsets, some of which may even exert tumor-suppressive functions. Deciphering the complex biology of CAFs is crucial for advancing CCA therapy. This review provides a thorough examination of the origins, functions, and pro-tumorigenic mechanisms of CAFs in the CCA TME, alongside a critical evaluation of advancements and obstacles in the development of therapies targeting CAFs.

## 1 Introduction

Cholangiocarcinoma (CCA) is an invasive, heterogeneous malignant tumor originating from the epithelial cells of the bile ducts (1). Its annual incidence is approximately 1–2 cases per 100,000 individuals, with a dismal 5-year overall survival of only 7%–20% (2, 3). While surgical resection is the only possible cure, many patients are diagnosed at the late stages of the disease, ruling out the possibility of curative surgery. Moreover, recurrence is common even among those who undergo resection (2–4). Current systemic chemotherapy and adjuvant regimens primarily serve to prolong survival in unresectable disease (5). Given the unsatisfactory outcomes of existing therapeutic strategies, it is imperative to develop novel treatments to improve survival, reduce recurrence, and overcome therapeutic resistance.

According to the clinical anatomical localization, CCA is stratified into intrahepatic (iCCA), perihilar (pCCA), and extrahepatic (eCCA) forms (1). The histogenesis of this malignancy is complex, with a spectrum of cellular origins proposed. These include cholangiocytes, hepatic progenitor cells, liver stem cells, and transdifferentiated hepatocytes found near portal tracts (6). Owing to its remarkable molecular heterogeneity, a comprehensive integration of genetic, transcriptomic, epigenetic, and molecular characteristics are essential for developing personalized treatment approaches.

Histologically, CCA is characterized by malignant epithelial cells embedded within a dense desmoplastic stroma enriched in fibroblasts, lymphatic vessels, and various immune cell populations. Although the functional contributions of stromal elements remain incompletely understood, recent studies have highlighted the critical role of the TME in promoting the progression and invasion of CCA (7–10). Notably, CAFs emerge as central players within this microenvironment. Investigations employing both *in vivo* and *in vitro* models have underscored the existence of heterogeneous CAFs subsets across desmoplastic malignancies, including CCA, with divergent tumor-promoting and tumor-suppressive properties (11–13). For example, inflammatory CAFs (iCAFs) recruit immunosuppressive cells; myofibroblastic CAFs (myCAFs) characterized by collagen deposition may increase the risk of recurrence by 2.1 times; while antigen-presenting CAFs (apCAFs) regulate T-cell responses. Recognition of this heterogeneity has shifted therapeutic paradigms and opened novel avenues for intervention. Therefore, advancing combination treatment paradigms compels a shift from focusing solely on cancer cells to targeting the dynamic cellular interactions within the TME, particularly those orchestrated by CAFs (12–15). This review provides an updated synthesis on the heterogeneity, biological functions, and interactions of CAFs in CCA, with emphasis on their mechanistic roles and therapeutic potential, aiming to highlight future opportunities for precision medicine.

## 2 CAFs in cholangiocarcinoma

**Figure 1:**
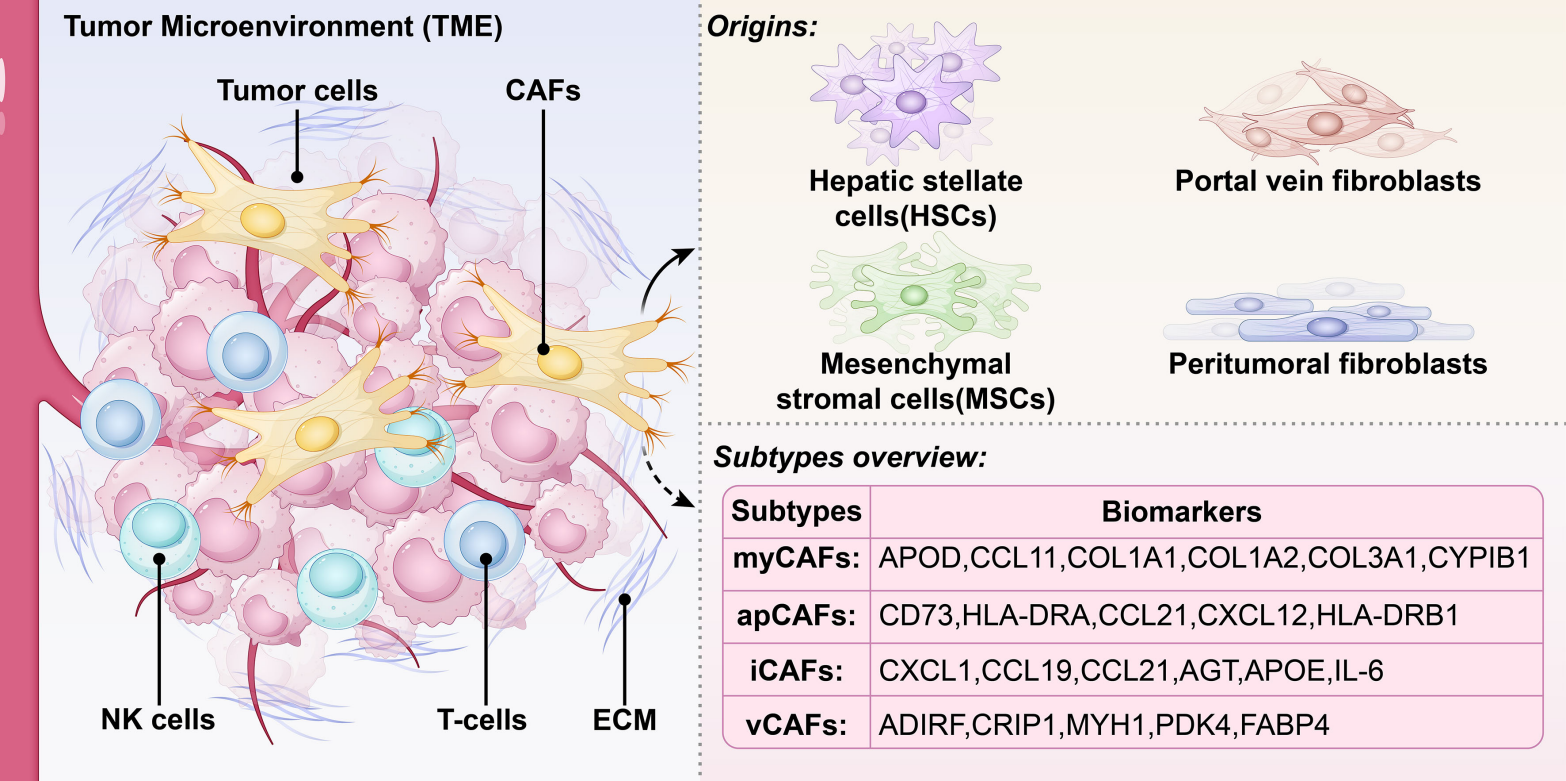
The TME in CCA and the origin and subtypes of its CAFs. The CCA TME is a complex, dynamic ecosystem regulating tumor progression (left side), comprising cellular components and non-cellular components. Cancer-associated fibroblasts within the TME originate from multiple cellular sources (upper right side), encompassing hepatic stellate cells (HSCs), portal vein fibroblasts, mesenchymal stromal cells (MSCs), and peritumoral fibroblasts. The lower right diagram illustrates several key CAF subtypes and their biomarkers, including myofibroblastic CAFs (myCAFs), antigen-presenting CAFs (apCAFs),inflammatory CAFs (iCAFs), and vascular CAFs (vCAFs).

CCA harbors a unique tumor microenvironment (TME) that dynamically regulates tumor progression, immune evasion, and therapeutic resistance through multiple mechanisms (16–18). The TME of CCA comprises a dynamic ecosystem of diverse elements, both cellular and non-cellular (**Figure 1** Left side). Within the TME, CAFs are a pivotal and heterogeneous constituent that engage in extensive crosstalk with various cell types, including malignant, immune, and endothelial cells (23, 24). Their ontogeny in CCA is multifaceted, deriving from multiple sources like hepatic stellate cells (HSCs), mesenchymal stromal cells (MSCs), portal fibroblasts and peritumoral fibroblasts (25–27) (**Figure 1**, Upper right side). The detection of specific activation markers, notably fibroblast activation protein (FAP) and platelet-derived growth factor receptor-α/β (PDGFR-α/β), provides valuable diagnostic utility for identifying the CAF population (11, 19, 20). By secreting various cytokines, growth factors, and ECM proteins, CAFs contribute to angiogenesis, immunomodulation, and fibrosis (21). Although traditionally regarded as tumor-promoting, CAFs have recently been shown to encompass transcriptionally distinct subsets with both pro- and anti-tumorigenic functions (22, 23). This complexity highlights the necessity of refining CAFs subtype characterization to enable effective therapeutic targeting in CCA.

## 3 Heterogeneity of CAFs

CAFs display profound heterogeneity in their cellular origin, transcriptomic features, and functional outputs, representing one of the most plastic cell populations within the TME. The application of scRNA-seq has resulted in the discovery of numerous transcriptionally unique CAF subpopulations in CCA **Table 1** (19, 20, 24). Although a definitive consensus on subset number and naming remains elusive, currently identified categories nevertheless encompass myofibroblastic CAFs (myCAFs), which demonstrate prominent extracellular matrix-depositing capabilities; inflammatory CAFs (iCAFs), which are enriched for transcripts of cytokines, chemokines, and growth factors; and antigen-presenting CAFs (apCAFs), characterized by high expression of MHC class II molecules and potential immunoregulatory capacity (12–15)(**Figure 1** Lower right side). In addition, more recently identified subtypes such as vascular CAFs (vCAFs) are associated with vascular niche support functions.Within the CCA TME, iCAFs or vCAFs are often the most abundant subsets. MyCAFs typically represent the second largest population and actively participate in matrix remodeling and fibrotic responses by producing ECM components such as collagen and fibronectin (12, 13, 15). Beyond the common myCAF and iCAF subtypes, studies have identified functionally specialized niche CAF subtypes. ApCAF is characterized by MHC class II molecule expression, suggesting potential antigen-presenting capabilities. MesCAFs typically originate from mesothelial cells and highly express markers such as mesothelin. CD10^+^GPR77^+^ CAFs have been shown to promote chemotherapy resistance by maintaining tumor stemness. Meanwhile, PDPN^+^ CAFs are closely associated with immune suppression and tumor metastasis (25, 26).

**Table 1:** More CAF subtypes and markers.

CAF subtypes	Primary biomarkers	Refs
myCAF	ACTA2/αSMA,TAGLN,PDGFRβ,COL1A1, HAS2, SERPINF1	(13, 15, 30)
iCAF	FAP, IL6, LIF, CXCL12, CCL11, HGF, RGS5	(13, 15, 30)
apCAF	MHC II (such as HLA-DRA), CD74	(13, 15, 30)
mesCAF	MSLN, UPK1B, UPK3B, GPM6A	(13, 15, 30)
vCAF	CCL8, GJA4, MHY11, MCAM, RGS5, IL-6	(13, 15, 30)
CD10^+^ GPR77^+^ CAF	CD10, GPR77	(26)
PDPN ^+^CAF	PDPN (Podoplanin)	(25)

The phenotypic plasticity of CAFs is a core characteristic. Recent studies demonstrate that biomechanical properties of the stroma, such as viscoelasticity, together with integrin-mediated adhesion, cooperatively regulate CAF differentiation states. Moreover, programmable hydrogel systems have been employed to direct patient-derived CAFs toward either myCAFs or iCAFs phenotypes by activating the JAK/STAT signaling pathway (27). Spatial multi-omics analyses have further revealed the spatial conservation of CAF subtypes. Tumor-adjacent s1-CAFs highly express ACTA2 and TGFB1, thereby promoting ECM remodeling and immune exclusion, while s4-CAFs, which are associated with tertiary lymphoid structures, express high levels of HLA-II molecules and chemokines, supporting anti-tumor immune responses (28).

The plasticity of CAFs is closely linked to their functional heterogeneity. While the majority of CAFs subsets promote tumor progression through matrix stiffening, angiogenesis, and immunosuppression, certain CAF populations may exert tumor-suppressive functions under specific conditions. For example, in hepatocellular carcinoma, LAMA4^+^ CD90^+^ endothelial CAFs induce senescence of CD8^+^ T cells, thereby suppressing anti-tumor immunity; conversely, targeting LAMA4 can reverse immunosuppression and enhance the efficacy of PD-1 blockade (29). This functional heterogeneity necessitates integrative approaches that combine multi-omics profiling, lineage-tracing, and functional assays. Such strategies are crucial to dissect the precise mechanisms through which discrete CAF subsets drive tumor progression and confer therapy resistance, thereby underpinning the development of precision interventions targeting CAFs.

## 4 Clinical relevance of CAFs

In the clinical practice of intrahepatic cholangiocarcinoma, the clinical value of CAFs is primarily reflected in four key dimensions. First, regarding CAF functional subtypes, myCAFs generate dense matrix barriers through extensive collagen fiber production, while iCAFs establish an inflammatory microenvironment conducive to tumor growth by continuously secreting cytokines such as CXCL12, HGF, and IL-6 (13, 31, 32). This functional differentiation directly influences therapeutic strategy selection. Second, regarding treatment resistance mechanisms, CAFs exert effects through three pathways: (1) The physical barrier constructed by myCAFs significantly reduces the effective concentration of chemotherapeutic drugs within tumor tissue; (2) Bypass signaling pathways provided by iCAFs, such as HGF/c-MET, enable tumor cells to evade drug-induced apoptosis; (3) Factors secreted by CAFs, such as CXCL12 and TGF-β, recruit immunosuppressive cells and inhibit T-cell function, limiting the efficacy of immunotherapy (33). Furthermore, as prognostic predictors, molecular subtyping based on CAF subtypes demonstrates superior predictive value compared to single biomarkers. For instance, a high iCAF profile correlates significantly with early recurrence and shorter overall survival, providing more precise tools for clinical prognosis assessment (10, 32). Finally, in clinical translation, therapeutic strategies have shifted from early nonspecific matrix clearance to precision modulation. Examples include using CXCR4 antagonists to block iCAF-derived CXCL12 signaling pathways or inducing CAF phenotypic reprogramming via vitamin D analogues(NCT02826486). These interventions targeting specific CAF subsets open new avenues for improving the current treatment landscape of cholangiocarcinoma.

## 5 Mechanisms by which cancer-associated fibroblasts mediate malignant phenotypes in cholangiocarcinoma

### 5.1 CAFs-mediated tumor proliferation and invasion

**Figure 2:**
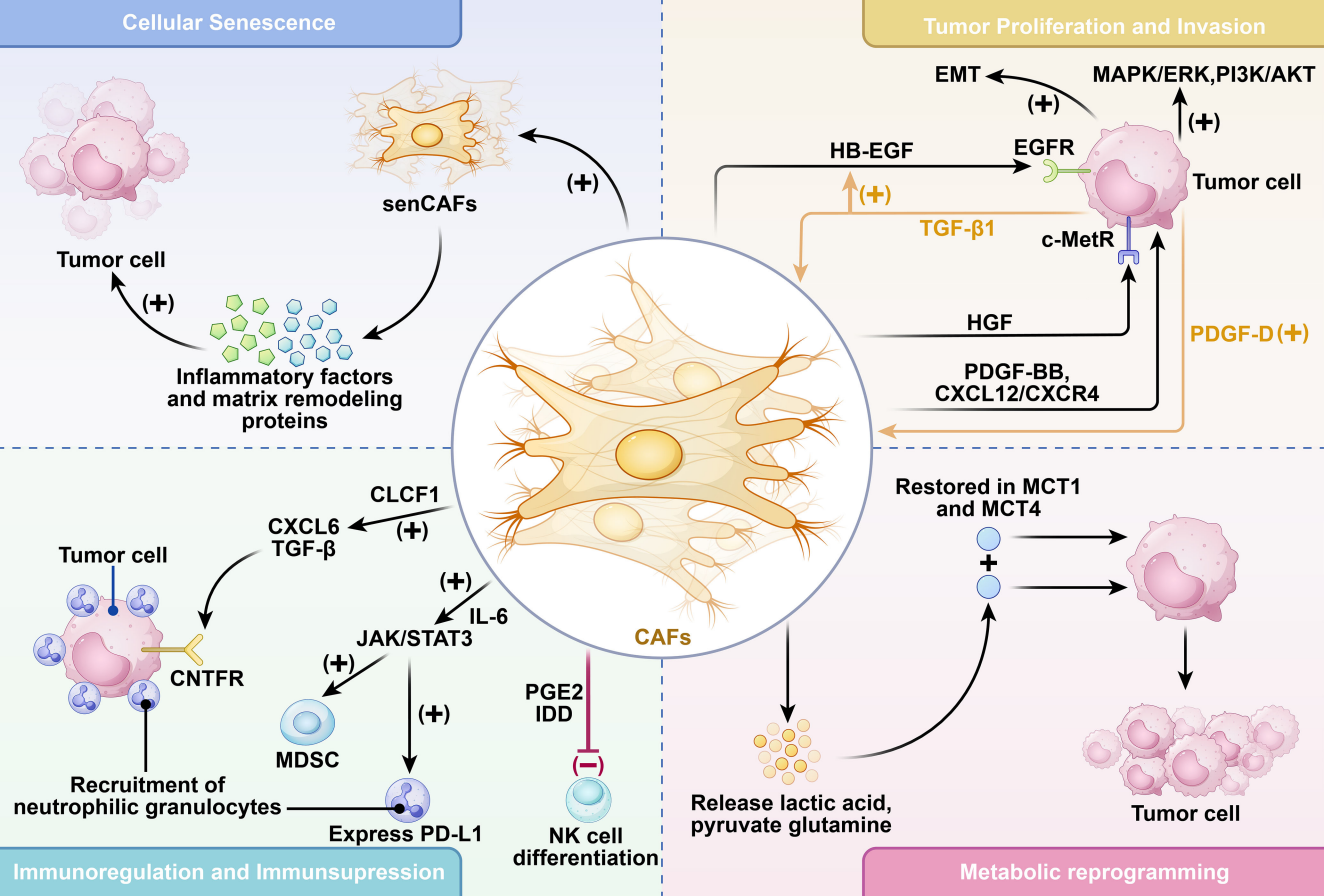
Mechanisms of CAF-mediated tumor progression and proliferation. CAFs participate in CCA progression through multiple mechanisms, exerting significant influence on tumor cell proliferation and invasion, immune regulation and suppression, cellular senescence, angiogenesis, and metabolic reprogramming. CAFs activate multiple signaling pathways to promote tumor cell proliferation by secreting growth factors. CAFs modulate the immunosuppressive microenvironment they generate, recruiting or suppressing various immune cell types to facilitate tumor cell immune escape. CAFs can undergo senescence, producing pro-inflammatory cytokines and matrix proteins that promote tumor cell growth. CAFs alter their own metabolic profiles, secreting substances like lactate that stimulate tumor cell proliferation. These findings collectively establish CAFs as master regulators of CCA initiation and progression, which provides a powerful rationale for developing targeted therapeutic interventions.

Within the desmoplastic tumor microenvironment of CCA, a highly intricate paracrine signaling network is established between cancer cells and CAFs, in which multiple growth factors and cytokines are exchanged in a reciprocal manner. This bidirectional communication is crucial in accelerating tumor growth and enhancing invasive behavior. Notably, CAFs release heparin-binding EGF-like growth factor (HB-EGF) as a pivotal paracrine signal. This ligand specifically engages the epidermal growth factor receptor (EGFR) displayed on malignant epithelial cells, initiating a cascade of downstream signaling events. (**Figure 2**) This receptor–ligand interaction not only augments tumor cell motility and invasion but also facilitates the induction of epithelial–mesenchymal transition (EMT) (34). In response to HB-EGF stimulation, malignant cells upregulate the secretion of transforming growth factor-β1 (TGF-β1). TGF-β1 subsequently acts back on CAFs to further upregulate HB-EGF expression, thereby forming a self-perpetuating, positive feedback loop that amplifies oncogenic signaling (34) (**Figure 2**) CAFs also secrete hepatocyte growth factor (HGF), which binds to the c-Met receptor on malignant cells. This interaction triggers downstream PI3K/AKT and MAPK/ERK signaling cascades (**Figure 2**). The activation of these cascades collectively enhances key malignant properties, including proliferation, survival, and evasion of apoptosis (13, 35, 36). A key secretory product of CAFs is platelet-derived growth factor-BB (PDGF-BB), which suppresses TRAIL-induced apoptosis by activating the Hedgehog signaling pathway, further promoting cancer cell viability (37) (**Figure 2**). Conversely, platelet-derived growth factor-D (PDGF-D) is primarily derived from the tumor cells, which activates RAC1/CDC42 and JNK pathways within CAFs, thereby stimulating their activation and migratory capacity (38). Another key axis is CXCL12/CXCR4 (**Figure 2**), whose activation results in enhanced ERK and PI3K/AKT signaling in tumor cells, directly linking to increased vascular invasion and poor clinical prognosis (39, 40). Beyond soluble factors, CAFs also exchange genetic material with tumor cells via exosomes, particularly microRNAs, further strengthening malignant phenotypes through post-transcriptional regulation (41). Taken together, these multifaceted interactions create a microenvironment that strongly favors tumor proliferation, migration, and invasion, representing multiple opportunities for therapeutic intervention.

### 5.2 CAFs in immunoregulation and immunosuppression

CAFs are not only structural components of the TME but also active regulators of immune responses, frequently skewing the immune landscape toward an immunosuppressive phenotype (42). Through the release of soluble factors and direct cell interactions, CAFs act as pivotal architects of the immunosuppressive niche by reprogramming the identity and activity of resident immune cells, thereby inducing a tolerogenic state. In hepatobiliary cancers, CAF-derived prostaglandin E2 (PGE_2_) and indoleamine 2,3-dioxygenase (IDO) directly suppress natural killer (NK) cell cytotoxic activity (43, 44), whereas interleukin-6 (IL-6) activates the JAK/STAT3 pathway in neutrophils, resulting in PD-L1 expression and enhanced differentiation of myeloid-derived suppressor cells (MDSCs) and alternatively activated M2 macrophages (45) (**Figure 2**). Moreover, CAFs express high levels of the transmembrane protein endosialin, which regulates the release of growth arrest-specific protein 6 (GAS6), thereby recruiting macrophages and inducing their differentiation toward the immunosuppressive M2 phenotype (46).

CAFs–tumor cell interactions further reinforce immunosuppression. For instance, CAFs-derived cardiotrophin-like cytokine factor 1 (CLCF1) engages ciliary neurotrophic factor receptor (CNTFR) on tumor cells, promoting the release of CXCL6 and TGF-β. These molecules, acting in a CXCR1/2-dependent manner, recruit neutrophils and polarize them into the pro-tumor N2 subtype, thereby facilitating tumor progression (47) (**Figure 2**). In CCA, bile acid–activated GPBAR1 signaling in CAFs induces the release of CXCL10, leading to neutrophil infiltration. Consequently, inhibiting this pathway potently potentiates the anti-tumor efficacy of PD-1 immune checkpoint blockade (48). Additionally, CAFs overexpress nicotinamide N-methyltransferase (NNMT), which promotes the accumulation of MDSCs. Pharmacological inhibition of NNMT restores CD8^+^ T-cell cytotoxicity and significantly improves responses to immunotherapy (49). Collectively, these findings underscore that CAFs function as master regulators of immunosuppression in the CCA TME, facilitating immune evasion and therapy resistance.

### 5.3 The role of CAFs in tumor metastasis

#### 5.3.1 CAFs and angiogenesis

Neovascularization is a key feature of CCA. Despite its overall low vascularization, CCA development requires establishing new vascular networks to support tumor growth (50, 51). In this process, CAFs directly promote lymphangiogenesis and tumor metastasis by secreting key factors such as VEGF-A and VEGF-C (52). Recent single-cell sequencing studies have further identified a vCAF subpopulation. This subset highly expresses markers such as IL-6 and RGS5, and promotes tumor progression through mechanisms including the IL-6/IL-6R axis and the exosomal miR-9-5p pathway (15).

#### 5.3.2 CAFs and lymphangiogenesis

CAFs play a pivotal role in driving lymphangiogenesis in CCA. PDGF-D secreted by CCA cells activates CAFs, which then upregulate VEGF-A and VEGF-C secretion via the ERK/JNK signaling pathway. These factors subsequently bind to VEGFR2 and VEGFR3 on lymphatic endothelial cells, promoting lymphatic vessel formation, increasing vascular permeability, and creating conditions for tumor cell lymphatic invasion (50, 53). *In vivo* experiments confirm that eliminating CAFs or blocking VEGFR signaling significantly inhibits tumor lymphangiogenesis and lymph node metastasis. Recent research has clarified the “CCA cells-PDGF-D-CAFs-VEGF/LECs” signaling axis, providing a theoretical basis for targeting the tumor microenvironment to suppress lymphatic metastasis in CCA (52).

#### 5.3.3 The role of CAFs in cancer stem cells

CAFs drive cancer stem cell properties by establishing complex paracrine networks. Key factors secreted by CAFs—including HGF, IL-6, folliculostatin-like protein 1 (FSTL1), and cardiotropic cytokine-like factor 1 (CLCF1)—activate multiple signaling pathways such as MET-ERK1/2, STAT3/Notch, TLR4/AKT/mTOR, and CLCF1/CXCL6-TGF-β autocrine circuits (54, 55), collectively promoting stemness marker expression and tumor stem cell self-renewal. This regulation exhibits highly complex bidirectional interactions: the CLCF1-induced cytokine circuit can activate CAFs through ERK1/2 signaling feedback, forming a self-sustaining microenvironment (47); simultaneously, CAFs promote tumorigenesis via the FOXQ1/NDRG1 axis and recruit additional CAFs through the pSTAT6/CCL26 pathway (56). Research further reveals that CAFs shape myeloid-derived suppressor cells via 5-lipoxygenase, thereby promoting stemness in intrahepatic cholangiocarcinoma (57).

### 5.4 CAFs and cellular senescence

In addition to their activated phenotypes, CAFs within the TME may undergo cellular senescence, forming a distinct subset termed senescent CAFs (senCAFs) (**Figure 2**). While senCAFs possess a reduced capacity for proliferation, they actively release a suite of senescence-associated secretory phenotype (SASP) factors, which consist of pro-inflammatory cytokines and ECM-remodeling agents like matrix metalloproteinases (MMPs). These secreted factors paradoxically promote tumor progression. In liver cancer research, senescent HSCs (the primary source of CAFs in iCCA) release a series of pro-cancer factors such as SHh and Wnt10b through their SASP. This activates key signaling pathways like Hedgehog and Wnt, thereby promoting the malignant transformation of hepatocytes (58). Similarly, in CCA, while chemotherapy-induced CAF senescence aims to suppress cancer cells, its SASP may paradoxically exacerbate inflammatory responses, ultimately leading to tumor recurrence. Research indicates that the senescent state of CAFs may correlate with specific markers, such as low expression of Caveolin-1 (CAV1), and is associated with tumor-infiltrating immune cells and PD-L1 levels, suggesting its potential as a prognostic indicator (59, 60). Therefore, delving into the triggering mechanisms of senescence in different CAF subpopulations, the composition of their SASP, and its dynamic changes is crucial for understanding tumor evolution and developing novel therapeutic strategies.

### 5.5 CAFs and metabolic reprogramming

To sustain the high metabolic demands of continuous tumor cell proliferation and dissemination, cancer cells undergo metabolic reprogramming. The fundamental metabolic shift in cancer cells is the transition from mitochondrial oxidative phosphorylation to aerobic glycolysis, known as the Warburg effect.

(61). In CCA, this reprogramming manifests as enhanced glycolysis and glutaminolysis, providing essential intermediates for biosynthetic processes (62, 63). CAFs also undergo metabolic adaptations, preferentially engaging in aerobic glycolysis and releasing metabolites such as lactate, pyruvate, and glutamine (**Figure 2**). These substances are shuttled into tumor cells through monocarboxylate transporters MCT1 and MCT4, thereby fueling oxidative metabolism and tumor growth (61, 64). High expression of MCT1 and MCT4 in CCA tissues further supports the notion that CAF-driven metabolic support constitutes a therapeutic vulnerability (65). In addition to glycolytic pathways, lipid metabolism and autophagy in CAFs are increasingly recognized as important contributors to TME remodeling. A more comprehensive understanding of these processes may provide critical insights into how metabolic interactions between CAFs and tumor cells mediate therapeutic resistance and tumor recurrence.

## 6 CAFs as therapeutic targets

Given the complex CAFs–stroma–immune interactions, targeting CAFs represents an attractive therapeutic strategy in CCA. Interventions aimed at disrupting aberrant CAFs-mediated signaling may reprogram the immunosuppressive TME and enhance anti-tumor immunity (19).

Research has revealed that the BH3 mimetic Navitoclax selectively eliminates CAFs within the tumor microenvironment by inhibiting Bcl-2/Bcl-XL and inducing mitochondrial apoptosis. This drug exploits the vulnerability of CAFs, which lack Mcl-1 expression and possess mitochondria in a “death-prepared state,” thereby suppressing the progression and metastasis of CCA.This approach offers a novel therapeutic strategy for treating CAF-rich solid tumors (66, 67).Additionally, preclinical studies implicate CAFs-derived matrix metalloproteinases (MMPs), TGF-β, and IL-6 as key drivers of immunosuppression and tumor progression (68). For instance, the TGF-β receptor I inhibitor galunisertib demonstrated anti-tumor efficacy in preclinical models (69).However, failed to improve overall survival when combined with gemcitabine/cisplatin in a phase II CCA trial (NCT02154646) (70). Targeting IL-6 with tocilizumab enhanced chemosensitivity and reduced metastasis in preclinical CCA models (71), though clinical evaluation in advanced biliary tract cancers (NCT04238715) remains preliminary. Similarly, HGF/c-Met inhibitors such as tivantinib failed to improve survival in advanced hepatocellular carcinoma (METIV-HCC, NCT01755767), raising caution for analogous strategies in CCA (72). Concurrently, a recent study also revealed that inhibiting the highly expressed SERPINE1/PAI-1 in CAFs can reverse the stromal barrier and enhance chemotherapy sensitivity, thereby enabling effective intervention in the tumor microenvironment (73).These findings highlight the limitations of single-pathway blockade and underscore the need for refined patient stratification, targeted delivery systems, and rational combination regimens.

## 7 Current status and future prospects

The central role of CAFs in shaping the desmoplastic TME of CCA has positioned them as promising therapeutic targets (16). scRNA-seq and bioinformatic analyses have revealed CAFs phenotypic heterogeneity, identifying both tumor-promoting and tumor-restraining subsets (19). Notably, studies based on human CCA specimens further confirm that specific CAF subtypes (such as myCAF and iCAF) are closely associated with patient prognosis and treatment response, highlighting their potential value in clinical practice (13).In translational research, intervention strategies targeting CAFs have evolved along multiple pathways. At the pharmacological level, researchers are exploring (1): direct depletion of pro-tumor CAF subpopulations, such as using antibody-drug conjugates targeting FAP (2); inhibition of key signaling pathways, such as employing TGF-β receptor inhibitors to block myCAF differentiation and subsequent matrix deposition (3); modulating their immune function, such as “rescuing” iCAFs from immunosuppression via CXCL12-CXCR4 axis inhibitors to enhance T-cell infiltration (13, 15). Recent research reveals that photothermal therapy (PTT) based on GIONFs, as an efficient physical treatment modality, directly disrupts dense physical barriers and immunosuppressive microenvironments. This approach pioneers a novel pathway for “physical-immune” regulation, successfully transforming tumors from an immune-cold state to an immune-hot state. It lays a solid foundation for synergistic enhancement with immunotherapies such as PD-1 inhibitors (74).While preclinical strategies such as depletion of specific CAFs populations or blockade of CAFs signaling have shown promise, clinical translation has been limited, likely due to CAFs plasticity and their intricate regulatory networks with tumor and immune cells (75).

Current challenges include the lack of reliable, specific surface markers for precise CAFs subtype discrimination, restricting the development of targeted therapies (75). Histological techniques and available antibodies remain inadequate for clinically actionable CAFs classification. Moreover, CAFs undergo rapid phenotypic drift *in vitro*, and existing models poorly recapitulate ECM composition and cell–cell interactions, creating discrepancies between preclinical and clinical findings (76). Additionally, the complexity of the TME and dynamic CAFs–stroma interactions necessitate development of more physiologically relevant models.

Future directions should include elucidation of CAFs–tumor metabolic symbiosis, particularly involving metabolites such as lactate, glutamine, and ketone bodies (77). Integration of spatial multi-omics and microbiome analyses may further reveal microbe–immune–CAFs interactions in CCA (78). Development of TME-responsive nanomedicine platforms and bispecific molecules targeting specific CAFs subsets or senescence-associated secretory phenotype (SASP) factors, in combination with immunotherapies, holds promise to overcome microenvironment-mediated resistance and provide novel therapeutic avenues for advanced CCA (79).
